# Negative Hits Hit Different

**DOI:** 10.21203/rs.3.rs-6857483/v1

**Published:** 2025-06-18

**Authors:** Arkadiy L. Maksimovskiy, Abigail Moline, Daniel G. Dillon

**Affiliations:** McLean Hospital; McLean Hospital; McLean Hospital

**Keywords:** Emotion, Memory, Retrieval, Valence, Arousal, Confidence

## Abstract

Neuroimaging of recognition memory reveals that the striatum responds more strongly to Hits (encoded stimuli recognized as old) vs. Correct Rejections (CRs: lures recognized as new), possibly because remembering old items is rewarding. If this is so, then Hits should elicit higher valence ratings than CRs for emotional and neutral stimuli. Alternatively, memory may interact with emotion such that while positive and neutral Hits drive valence up, negative Hits drive valence down (relative to CRs of the same type). We investigated this by analyzing data from 47 healthy participants who encoded negative, neutral, and positive pictures, completed a recognition memory test, and rated the emotions elicited by each picture. Valence ratings were higher for neutral and positive Hits vs. CRs but only positive FAs (FAs; falsely recognized lures) elicited higher valence ratings than CRs. Strikingly, negative Hits elicited lower valence ratings than negative CRs. The impact of memory on subjective experience thus varied: for neutral pictures, accurate memory enhanced valence; for positive pictures, perceived oldness (accurate or not) boosted valence; and for negative pictures, accurate memory reduced valence. The impact of memory retrieval on subjective experience thus depends on the emotional nature of the memoranda.

## Introduction

Research on emotional memory has played a foundational role in the emergence of affective science. After decades of relatively modest work, investigations of fear conditioning and amygdala function in non-human animals (e.g., Davis & Astrachan, 1978; McGaugh, 1983; LeDoux et al., 1984) gradually inspired similar research in humans, and what began as a trickle is now a torrent: early studies established a key role for the amygdala in human fear conditioning but also in the encoding and consolidation of negative episodic memories (LaBar & Cabeza, 2006), and voluminous research in the years since has examined, among many other topics, differences between positive and negative memories (Williams et al., 2022), and the impact of emotion regulation, personality, and social factors on emotional memory (Dolcos et al., 2017). In short, the impact of emotion on memory is now well-established. Our aim in this analysis was to contribute to that large literature by asking a different question: what is the empact of memory on emotion?

Our question was inspired by an interesting finding from functional magnetic resonance imaging (fMRI) studies of recognition memory: successful memory retrieval robustly engages the brain’s reward circuitry (Scimeca & Badre, 2012). Specifically, the striatum—a region central to reward processing (Delgado, 2007)—is activated more strongly by *Hits* (correctly recognized “old” stimuli from encoding) compared to *correct rejections* (CRs; lures accurately identified as new). The psychological mechanisms underlying this result are not entirely clear, but Han et al. (2010) proposed that the effect emerges because, by remembing old material, the participant has achieved what they take to be the implied goal of the task—namely, remembering studied items. In support of this interpretation, Han et al. (2010) showed that if Hits were incentivized with monetary rewards then the size of the Hits > CRs striatal effect increased, but if CRs were incentivized then the effect flipped: the striatum responded more strongly to CRs vs. Hits. The striatum’s sensitivity to the current goal implies that, in typical recognition memory experiments, participants likely adopt the goal of remembering old items (as opposed to balancing memory for old items vs. ability to reject lures, for example), and achieving that goal elicits striatal activation.

This clever experiment led to two questions. First, would memory retrieval have detectable effects on ratings of current emotional experience? If the inferences made by Han et al. (2010) are correct, then—for neutral items—it seemed that Hits should elicit more positive emotional responses than CRs; this could be assessed by asking participants to rate their responses to each stimulus for valence (pleasantness) after the memory test. Second, do any effects of memory on subjective experience vary with the emotional nature of the memoranda? The stimuli used by Han et al. (2010)—and by most studies of recognition memory—were neutral, but if goal attainment is key then one might expect similar results for neutral, negative, and positive stimuli. In other words, although such stimuli clearly differ strongly on valence, within each stimulus category one might expect higher valence ratings for Hits vs. CRs, because in each case remembering old items satisfies the participant’s goal. Alternatively, the effect could vary depending on the stimuli: successfully remembering positive and neutral items might be pleasurable, whereas retrieving negative memories might be unpleasant—even relative to successfully rejecting negative lures. Our main goal was to contrast these two hypotheses: a first one in which Hits always feel more pleasant than CRs, versus a second in which the effect depends on the emotional nature of the memoranda—positive and neutral Hits feel better than CRs, whereas negative Hits feel worse.

We contrasted these possibilities using data from an experiment in which participants encoded negative, neutral, and positive pictures, completed a surprise recognition memory test 24 hours later, and then rated their emotional reaction to each picture for valence and arousal. This design allowed us to classify recognition responses as Hits, CRs, False Alarms (FAs; new items endorsed as old), and Misses (old items endorsed as new), and then relate these response types to valence ratings.

We also examined arousal and confidence. Arousal is considered orthogonal to valence (i.e., it can be high for negative and positive stimuli), but more extreme valence ratings typically correlate with higher arousal ratings (Lang, 1995). This correlation between valence and arousal allowed us to contrast two more alternatives. If the first hypothesis is correct and Hits elicit more positive valence ratings than CRs across all picture types, then valence ratings—and presumably arousal ratings as well—would be more extreme (closer to the upper limit) for positive and neutral Hits vs. CRs, but valence ratings—and presumably arousal ratings—would be less extreme (further from the lower limit) for negative Hits vs. CRs. Consequently, a *PictureType* x *ResponseType* interaction would be expected for arousal ratings (i.e., higher arousal for Hits vs. CRs for positive and neutral pictures, but not negative pictures). By contrast, if the second hypothesis is correct and valence is higher for Hits vs. CRs for positive and neutral pictures, but lower for Hits vs. CRs for negative pictures, then valence ratings would be more extreme for Hits vs. CRs across picture types, and presumably the same would be true for arousal ratings. In this case, analysis of arousal ratings should yield a main effect of *PictureType*. Finally, examining confidence allowed us to explore whether stronger memories, associated with higher confidence, elicited more intense effects on emotional experience than weaker memories.

## Method

### Participants

Forty-seven healthy adults were recruited through online advertising. These participants are part of a larger electroencephalography (EEG) study examining the impact of depression on memory—the study is onging, and the data presented here are from healthy controls and are focused on questions distinct from those asked in the larger study. Because we generated the analysis ideas when the larger study was underway, the sample size for this analysis was not planned; we simply analyzed all the healthy control data we had available. Participants were 18–55, right-handed, non-smokers, and free of psychiatric or neurological conditions; they provided written consent to a protocol approved by the Mass General Brigham Institutional Review Board (“Emotional Information Processing in Depression”; #2021P000932). Eligible individuals completed an online diagnostic interview followed by two in-person sessions at McLean Hospital, and were compensated $25/hour.

### Stimuli

Pictures came from a separate study designed to generate pairs of closely matched images (Maksimovskiy et al., 2022), as such matching is difficult to achieve with commonly used picture sets. Three hundred pictures were selected: 50 positive, 50 negative, and 50 neutral pairs. We intended positive and negative pictures to differ on valence but not arousal, whereas neutral pictures were meant to differ from emotional pictures on valence and arousal. Before examining how memory retrieval affected valence and arousal ratings in the current study, we examined the impact of picture type on these ratings to confirm that the intended emotional responses were elicited (see Results).

In the current study, the oddball paradigm (McCarthy & Donchin, 1976) served as the encoding task. For each participant, one picture per pair served as an oddball target, with the other used as a recognition lure. Six hundred diffeomorphed pictures were frequent oddball “standards”. The diffeomorphing procedure, implemented in MATLAB (Mathworks, Natick MA), transformed the pictures into abstract shapes while preserving luminance and color composition (Stojanoski & Cusack, 2014). As per the oddball paradigm, targets (20% of stimuli) were rare relative to standards (80% of stimuli).

### Procedure

Eligible individuals completed the Mini International Neuropsychiatric Interview (MINI; Sheehan et al., 1998) online to confirm the absence of psychiatric disorders. During the first in-person session, participants were fitted with a 96-electrode EEG cap; EEG data will be presented elsewhere and are not further discussed. They next watched a 10-minute neutral video before completing the oddball task on a desktop computer. On each trial, a target or standard was shown (2,000 ms), followed by a jittered inter-trial interval (500–1,500 ms). Participants responded with their right hands using a keyboard, pressing the left arrow for pictures and the right arrow for standards. They completed a brief practice block followed by 750 trials, with a break halfway through. The task were conceptualized as including 50 15-trial sequences, with each sequence including three pictures—one of each valence—and 12 standards, thus ensuring that every picture type appeared with equal frequency in the beginning, middle, and end of the task.

Participants returned the next day for a surprise memory test, in which they viewed the 150 encoded pictures and 150 lures (shown in random order), indicated whether each picture was old (from encoding) or new (a lure), and rated their confidence on a 3-point scale (low, medium, high). The test was self-paced and took about 20 minutes.

Next, participants rated their emotional response to each picture using Affective Sliders (Betella & Verschure, 2016). Two sliders were positioned next to each picture, with the top slider for arousal and the bottom slider for valence. Participants made their ratings by placing a cursor along a continuous line without visible numerical markers. Although no scale values were displayed during the task, responses were recorded internally on a 1–100 scale for analysis. At the endpoints of each slider, stylized emoticons depict bipolar affective states: unhappy to happy for valence, and sleepy to wide-awake for arousal. Ratings were self-paced. All tasks were coded in PsychoPy (Peirce et al., 2019).

### Data Analysis

Analysis was conducted using R (version 4.4.1; R Core Team, 2021) and Python (version 3.12.5). Cleaning and plotting used Jupyter Notebooks (Kluyver et al., 2016) and the *matplotlib* (Hunter, 2007), *numpy* (Harris et al., 2020), *pandas* (Pandas Development Team, 2024), and *seaborn* (Waskom, 2021) packages. R analysis used *afex* (Singmann et al., 2020), *effsize* (Torchiano, 2020), *emmeans* (Lenth, 2022), *lmerTest* (Kuznetsova, Brockhoff, & Christensen, 2017), and *tidyverse* (Wickham et al., 2019). This analysis was not pre-registered.

We report how we determined our sample size, all data exclusions (if any), all manipulations, and all measures in the study.

Encoding response accuracy was analyzed to confirm that participants were attentive. We then ran repeated-measures ANOVAs to analyze the effect of *PictureType* (negative, neutral, positive) on *d*’ and *c*—signal detection measures of discriminability and response bias (Stanislaw & Todorov, 1999), respectively—to confirm that memory was adequate.

Next, we used trial-level linear mixed-effects models to examine relationships between *RecognitionResponse* (Hits, Misses, CRs, FAs) and valence ratings. Separate models were run for each picture type, with valence ratings as the dependent variable and *RecognitionResponse* as the independent variable. To account for individual differences and variation in the memorability of different pictures (Isola et al., 2014), picture names and subject IDs were included as random effects (valence ~ recognition_response + [1|subject] + [1|picture_name]). Similar models were run to examine the impact of memory on arousal ratings. Finally, we used linear regression to determine whether the effect of memory on emotional ratings varied with confidence. To streamline this analysis, we used Hit minus CR difference scores as the dependent variable, excluded neutral pictures, and used *PictureType* and *Confidence* (plus their interaction) as independent variables (e.g., Hit-CR_valence ~ picture_type * confidence).

#### Transparency and Openness

This study was not preregistered. However, we report how we determined our sample size, all data exclusions, manipulations, and measures in the study, following the JARS guidelines (Appelbaum et al., 2018). All data, analysis code, and research materials are publicly available at th*e Open Science Framework* (*OSF*; ttps://osf.io/dbstf/?view_only=e45acc5450534c76906923bc0ae25feb).

## Results

### Participants

Demographic data are in Table 1.

### Encoding

Mean±SD encoding accuracy was exceptionally high (99.1±1.4%).

### Memory

The effect of *PictureType* on *d’* was significant, *F*(2, 92) = 41.7, *p* < .001, ${\eta^{2}}_{\text{G}}=0.16$. Pairwise comparisons showed that *d’* was higher for negative pictures (1.34±0.67) vs. both neutral (0.74±0.46) and positive (1.11±0.53) pictures, *ts* > 3, *ps* < .02; *d’* was also higher for positive vs. neutral pictures, *t*(46) = 6.6, *p* < .001. The effect of *PictureType* on *c* was also significant, *F*(2, 92) = 7.20, *p* = .002, ${\eta^{2}}_{\text{G}}=0.04$.Pairwise tests indicated that *c* was lower for negative pictures (0.22±0.35) vs. both neutral (0.38±0.37) and positive (0.34±0.27) pictures, *ts* < −2.8, ps < .02. *c* did not differ reliably for neutral and positive pictures, *t*(46) = 1.09, *p* = .84. To summarize, discriminability was generally good—best for negative pictures and worst for neutral pictures—and participants showed a bias to respond “new” (all values of *c* > 0) that was weakest for negative pictures. These analyses confirmed that memory was sufficiently accurate to permit examination of its effect on subjective experience.

### Pictures Elicited Expected Emotions

Before considering the impact of memory on emotion, we examined the effect of *PictureType* on valence and arousal ratings to confirm that pictures elicited the expected emotions. The ANOVA on valence ratings was highly significant, *F*(2, 92) = 380.28, *p* < .001, ${\eta^{2}}_{\text{G}}=.86$. Valence ratings were lowest for negative pictures (20.3 ± 17.0), intermediate for neutral (47.8 ± 11.8), and highest for positive pictures (75.7 ± 16.7). All pairwise comparisons were significant, *ps* < .001.

The ANOVA on arousal ratings was also significant, *F*(2, 92) = 96.75, *p* < .001, ${\eta^{2}}_{\text{G}}=.52$. As intended, arousal levels were similar for negative (65.3±24.6) and positive (62.1±22.4) pictures, but lower for neutral pictures (24.5±23.4). Pairwise comparisons confirmed no difference between negative and positive pictures, *t*(46) = 1.30, *p* = .603, *d* = 0.14, but significant differences between negative and neutral pictures, *t*(46) = −10.45, *p* < .001, *d* = 1.7, and between positive and neutral pictures, *t*(46) = −11.78, *p* < .001, *d* = 1.64.

### Effect of Memory on Valence Ratings

**Figure 1:**
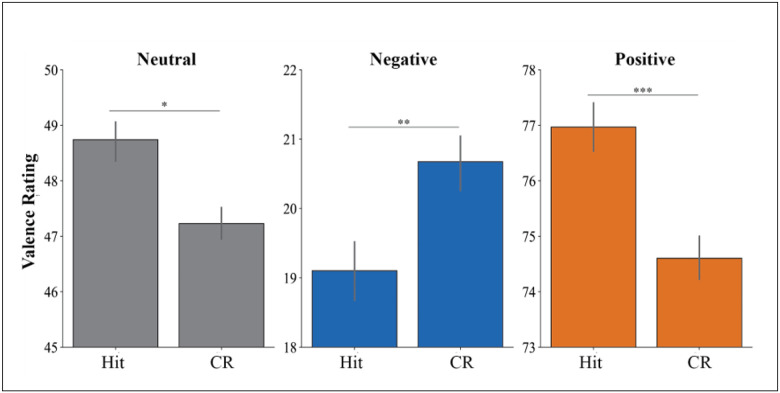
Valence Ratings for Hits and Correct Rejections *Note*. The y-axes differ across panels to reflect the distinct range of valence ratings for neutral, negative, and positive pictures. Error bars represent SEM.

Figure 1 shows that the impact of memory on valence ratings varied by picture type. For neutral and positive pictures valence was higher for Hits vs. CRs, but for negative pictures valence ratings were lower for Hits vs. CRs.

A linear mixed model for netural pictures confirmed higher valence ratings for Hits vs. CRs (*b* = 1.01, *SE* = 0.36, *z* = 2.84, *p* = .005, *d* = 0.12). No differences were observed between Hits and FAs (*b* = −0.50, *SE* = 0.48, *z* = −1.03, *p* = .304, *d* = 0.05) or between FAs and CRs (*b* = 0.52, *SE* = 0.47, *z* = 1.10, *p* = .271, *d* = −0.05).

For positive pictures, Hits again received higher valence ratings than CRs (*b* = 1.80, *SE* = 0.43, *z* = 4.18, *p* < .001, *d* = 0.21). No difference emerged between Hits and FAs (*b* = −0.53, *SE* = 0.66, *z* = −0.80, *p* = .426, *d* = −0.17), but FAs received higher valence ratings than CRs (*b* = 2.33, *SE* = 0.65, *z* = 3.58, *p* < .001, *d* =0.38).

For negative pictures, Hits received lower valence ratings than CRs (*b* = −0.94, *SE* = 0.45, *Z* = −2.10, *p* = .036, *d* = −0.18). No differences emerged between Hits and FAs (*b* = −0.11, *SE* = 0.69, *z* = −0.16, *p* = .873, *d* = −0.14) or between FAs and CRs (*b* = 0.83, *SE* = 0.69, *z* = 1.20, *p* = .230, *d* = 0.01). Descriptive data are in Table 2^1^.

### Arousal

**Figure 2:**
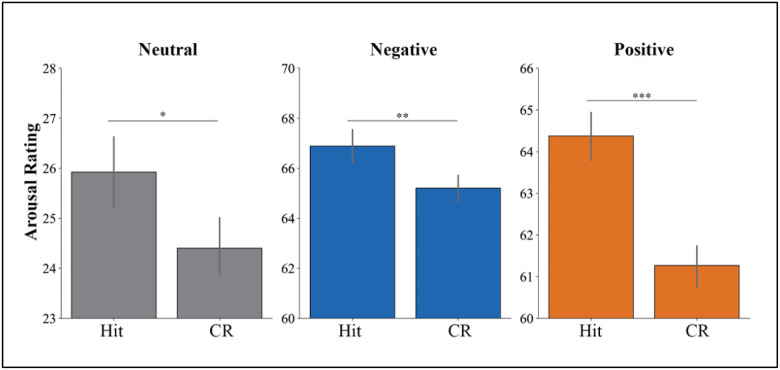
Arousal Ratings for Hits and Correct Rejections *Note*. The y-axes differ across panels to reflect the distinct range of arousal ratings for neutral, negative, and positive pictures. Error bars represent SEM.

Figure 2 shows that Hits were more arousing than CRs for neutral pictures (*b* = 1.90, *SE* = 0.52, *Z* = 3.64, *p* < .001, *d* = 0.08), positive pictures (*b* = 2.69, *SE* = 0.51, *z* = 5.25, *p* < .001, *d* = 0.18), and negative pictures (*b* = 1.53, *SE* = 0.55, *z* = 2.78, *p* = .005, *d* = 0.1).

### Confidence

**Figure 3:**
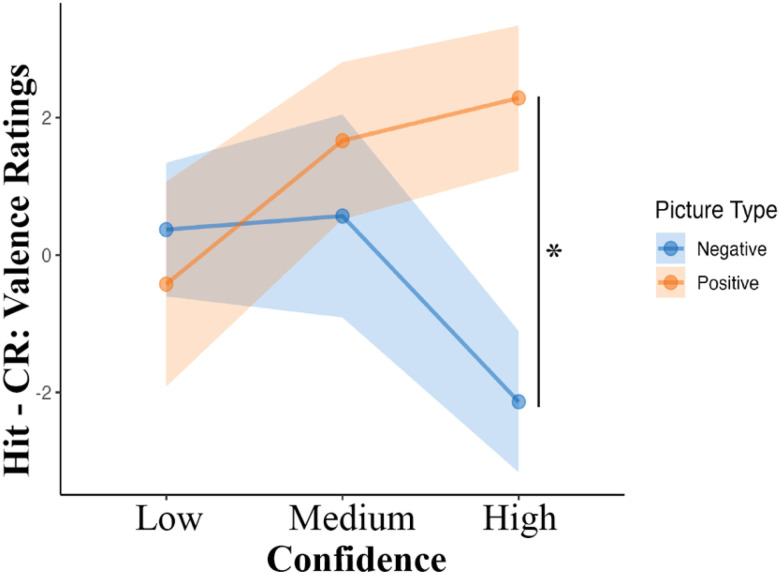
Confidence Modulates the Impact of Retrieval Success on Emotional Experience *Note*. Shaded regions indicate SEM.

Figure 3 shows that confidence modulated the strength of the Hit–CR effect on valence ratings elicited by emotional pictures (neutral pictures were excluded from this analysis). This impression was supported by a significant *PictureType* x *Confidence* interaction (*b* = 5.22, *SE* = 2.55, *t*(257) = 2.04, *p* = .042, ${\eta^{2}}_{\text{G}}=0.02$). For high confidence responses, the Hit-CR subtraction yielded higher valence ratings for positive vs. negative pictures (*b* = 4.42, *SE* = 1.75, *t*(257) = 2.53, *p* = .01, *d* = 0.62). This effect was not significant, however, for medium (*b* = −1.1, *SE* = 1.74, *t*(257) = −0.63, *p* = 1, *d* = 0.12) or low confidence responses (*b* = 0.79, *SE* = 1.86, *t*(257) = 0.43, *p* = 1, *d* = 0.09).

## Discussion

Inspired by findings from neuroimaging, we conducted this analysis to determine (1) whether the act of successfully retrieving neutral memories (Hits) elicited more pleasant subjective experience than correctly rejecting neutral lures (CRs), and (2) whether any effect of Hits vs. CRs on valence ratings observed for neutral pictures would be similar or different for positive and negative pictures. On the first point, there was a reliable increase in valence for neutral Hits vs. CRs. On the second point, the same pattern of results was observed for positive pictures, but here the boost in valence ratings extended from Hits to FAs. More importantly, the effect reversed for negative pictures: negative Hits elicited lower valence ratings than negative CRs. We reasoned that this pattern of results—where Hits are associated with more extreme positive and negative valence rating than CRs—would be paralleled by a main effect of *PictureType* on arousal ratings, and indeed this was also observed: across all picture types, Hits were more arousing than CRs. When the impact of confidence on valence ratings was examined, we found that the Hit > CR difference for negative vs. positive pictures was only reliable for high confidence responses. Overall, the impact of memory retrieval on subjective experience appears to depend on the emotional nature of the memoranda and is most apparent for strong memories.

### The Impact of Memory Retrieval on Subjective Experience Varies by Picture Type

Using neutal words as stimuli, Han et al. (2010) observed significant caudate activation in the Hits > CRs contrast, a result seen in several other studies (e.g., Scimeca & Badre, 2012). By manipulating monetary payoffs, they demonstrated that this activation is sensitive to goal attainment: when participants could earn rewards for Hits, the Hits > CR difference in caudate activation increased, but when participants could earn rewards for CRs, then the caudate showed the opposite effect (i.e., stronger activation for CRs vs. Hits). Given these results, and because the caudate is a striatal region that receives strong dopamine projections (Haber & Knutson, 2010), Han and colleagues reasoned that the Hit > CR contrast elicits caudate activation in recognition memory experiments because participants adopt the goal of remembering old item as opposed to balancing Hits with CRs.

Han and colleagues made no claims about a link between caudate activation and subjective experience, but a relationship between activation in the brain’s reward network and improved subjective experience seemed plausible, and the current results support this idea by confirming higher valence ratings for neutral Hits vs. CRs. Nevertheless, results from other picture types indicate that “goal attainment” is not sufficient to explain the current pattern of valence ratings, for two reasons. First, for positive pictures it was not necessary for participants to achieve the implied goal: valence ratings were boosted for positive FAs and for positive Hits relative to CRs, implying that simply believing the goal had been achieved was enough. Second, the effect flipped for negative pictures: negative Hits elicited lower valence ratings than negative CRs. In other words, achieving the presumed goal on negative trials did not make participants feel better, it made them feel worse. While none of these results challenge the inferennces drawn by Han et al. (2010), which were strictly about caudate activation in response to neutral memories, they do indicate that a more nuanced explanation is needed to explain why retrieving memories of different types has different effects on subjective experience.

The current results are too limited to provide clear insight into this issue, but they seem consistent with known differences in the impact of negative and positive emotion on subsequent memory (Bowen et al., 2018; Kensinger, 2007; Williams et al., 2022), and on cognition in general (Fredrickson, 2001; but see Sung & Yih, 2016). Evidence from several studies indicates that, overall, negative memories tend to be encoded with more sensory detail than positive memories, and at retrieval this sensory detail is recapitulated (for review, see Bowen et al., 2018). By contrast, positive memories typically include fewer item-level details, but—perhaps as a consequence—they seem to enter into associations more easily (e.g., Madan et al., 2019). Their retrieval has also been shown to be intrinsically rewarding (Speer & Delgado, 2014) and capable of repairing negative moods (Joormann & Siemer, 2004). And although experimental support for this position is mixed (e.g., Sung & Yih, 2016), the broaden-and-build theory (Fredrickson, 2001) proposes that positive emotions widen attention, promote connections between disparate ideas, and facilitate creativity and open-mindedness. Whereas intense negative emotions are associated with focused attention, narrower thinking, and more concrete reasoning. We do not have sufficient data to directly link these possibilities to the current results, but it is plausible that vividly recalled negative memories accompanied by numerous visual details could yield the most unpleasant experience, whereas, by encouraging a broader frame of mind, positive memory retrieval could cause the valence boost to extend from Hits to FAs. Additional research would be needed to test these speculations.

### Arousal and Confidence

The emotional memory literature has historically emphasized the key role of arousal, and for good reason: arousal at encoding is a potent driver of amygdala activation, amygdala modulation of hippocampal function, and the strength of subsequent memory via its effects on consolidation (LaBar & Cabeza, 2006; Dolcos et al., 2017; Mather & Sutherland, 2011; Phelps, 2006). Reflecting the well-established link between emotional arousal and enhanced memory discrimination (e.g., Mather & Sutherland, 2011), we observed that d’ was higher for negative and positive pictures compared to neutral pictures. Most importantly for present purposes, we also found a complementary effect: successful retrieval of old memories was experienced as more arousing than correctly rejecting lures. This effect held across all picture types, even though neutral pictures were inherently much less arousing than negative or positive pictures. We interpret the arousal result as in line with the findings for valence ratings: for all picture types, Hits elicited more extreme valence ratings than CRs, and since arousal is typically higher for more extremely valenced positive and negative emotional states, it follows that arousal should be consistently higher for Hits vs. CRs. In other words, Hits seem to amplify the emotional effects of the memoranda: positive Hits are experienced as especially pleasant and arousing, whereas negative Hits are experienced as especially negative and arousing. Perhaps because we analyzed data from healthy participants who typically possess an optimistic bias (Sharot, 2011), neutral Hits were also experienced as more pleasant and more arousing than neutral CRs. It remains to be seen whether these patterns would be observed in samples with less rosy views of life (e.g., depressed adults).

The data also indicated that memory strength is key. Specifically, when the valence ratings difference between Hits and CRs was compared for positive vs. negative pictures, the comparison was only significant for highly confident responses. Because confident memories may be experienced as especially vivid and emotionally arousing (Talarico & Rubin, 2003), this effect seems to dovetail with the interpretation offered earlier for the impact of negative Hits on valence: intense retrieval of sensory details may be what amplifies negative emotion for Hits. It is less clear whether this account can also explain responses to positive pictures. For example, although positive memories are generally less vivid than negative ones, this may not hold for high-confidence memories. In such cases, retrieving vivid sensory details could enhance the emotional impact of positive memories—just as it appears to reduce the emotional impact of negative ones. It is critical to remember, however, that these shifts in valence occurred within the context of a recognition test, where participants were always re-exposed to the stimulus. As a result, the effect of memory on emotional experience at retrieval—as well as the picture rating task—may be influenced by the act of viewing the image again. Understanding the specific ways in which re-exposure shapes emotional responses is an important direction for future research.

### Limitations

This analysis has several limitations. First, these analyses are exploratory in nature. The data come from an ongoing study primarily focused on other questions (i.e., the impact of depression on EEG responses during the encoding and retrieval of positive memories), and we took the opportunity to examine the available control data after the current research questions emerged. While the findings offer a promising starting point, replication and future studies specifically designed to address these questions would strengthen the conclusions. In particular, we infer that the rating differences are driven by the impact of memory; however, we lacked baseline ratings for a subset of pictures. Ideally, participants would provide ratings both before and after the memory test to track changes as a function of memory status. Second, although the results were visually apparent and statistically reliable, they were associated with modest effect sizes; methods that could boost effect size would be helpful. Third, we used an oddball paradigm as our encoding task due to goals of the larger study, but the oddball task is fairly uncommon in memory research. Because pictures were presented infrequently, this task minimizes the possibility for the emotional response elicited by one picture to influence the response to a subsequent picture. Nevertheless, future work could assess whether our findings extend to more conventional encoding tasks. Finally, it is unclear if the results would generalize to participants with psychiatric conditions known to affect emotional memory. In particular, we are interested to examine this possibility in depressed individuals, who show positive memory deficits (Burt et al., 1995; James et al., 2021) and whose negative self-concept (e.g., Beevers et al., 2019; Collins & Winer, 2024) might weaken the perceived oldness effect for positive pictures but increase it for negative pictures (i.e., depressed adults might show low valence ratings for negative Hits and negative FAs, while only showing a valence boost for positive Hits).

## Conclusion

Collectively, our findings demonstrate that the impact of memory on subjective experience depends on the emotional nature of the memoranda. In our healthy sample, believing a positive picture was old—whether or not that belief was accurate—was sufficient to boost valence ratings relative to positive CRs. By contrast, accurately retrieved negative pictures—Hits—intensified negative affect, driving valence down relative to negative CRs. Memory strength amplified these effects: indeed, they were only statistically reliable for highly confident responses. In short, memory can affect emotional experience and the result is not uniformly pleasant: retrieving positive memories (accurately or not) feels good, but accurately retrieving negative memories feels worse than rejecting novel negative items.

## Figures and Tables

**Table 1:** Participant Demographics

Variable	M (SD) or Frequency
Age (years)	30 (10.7)
Education (years)	16.5 (2.2)
*Sex*	
Female	31 (66%)
Male	16 (34%)
*Racial Identity*	
White	28 (59.6%)
Black/African American	3 (6.4%)
Asian	17 (36.2%)
Multiracial	—
Other	—
*Ethnicity*	
Hispanic/Latinx	5 (10.6%)
Non-Hispanic/Latinx	42 (89.4%)
*Household Income*	
Less than $10,000	4 (8.5%)
$10,000–$25,000	1 (2.1%)
$25,000–$50,000	14 (29.8%)
$50,000–$75,000	5 (10.6%)
$75,000–$100,000	4 (8.5%)
More than $100,000	19 (40.4%)

*Note*. For racial identity, participants may have selected more than one category; dashes (“—”) indicate categories for which no data were provided.

**Table 2:** Mean (SD) Valence Ratings

Picture Type	Response Type			
	*Hit*	*Miss*	*FA*	*CR*
Neutral	48.74 (12.14)	47.56 (11.49)	48.01 (11.72)	47.23 (11.90)
Positive	76.97 (15.93)	74.45 (17.25)	78.80 (15.50)	74.60 (17.00)
Negative	19.10 (16.96)	22.48 (17.73)	19.23 (17.22)	20.67 (16.63)

*Note*. FA = false alarm, CR = correct rejection

## Data Availability

Data and code are available on the Open Science Framework at the following link: https://osf.io/dbstf/?view_only=f820c02b63c84a8e96ee63f0dd003257

## Footnotes

1. To confirm the robustness of these trial-level analyses, we also conducted a more traditional repeated-measures ANOVA on subject-level mean ratings for valence. Results were consistent with the trial-level models, revealing a *PictureType* x *Response Type* interaction, F(6, 276) = 10.79, p < .001.
